# Vesical perfusion volume and internal iliac pressure during double balloon-occluded arterial infusion chemotherapy for bladder cancer

**DOI:** 10.1186/s41747-025-00620-y

**Published:** 2025-08-11

**Authors:** Kiyohito Yamamoto, Kazuhiro Yamamoto, Hiroshi Juri, Haruhito Azuma, Keigo Osuga

**Affiliations:** 1 Department of Diagnostic Radiology, Osaka Medical and Pharmaceutical University, Takatsuki-City, Osaka Japan; 2 Department of Urology, Osaka Medical and Pharmaceutical University, Takatsuki-City, Osaka Japan

**Keywords:** Angiography (digital subtraction), Arterial pressure, Perfusion, Radiology (interventional), Urinary bladder neoplasms

## Abstract

**Background:**

This study investigated the correlation between decreased internal iliac arterial blood pressure (IIABP) and blood perfusion volume within the vesical artery region during double-balloon-occluded arterial infusion chemotherapy (D-BOAI) for invasive bladder cancer, utilizing two-dimensional perfusion angiography (2D-PA).

**Materials and methods:**

Sixteen patients were enrolled in this study. A double-balloon catheter was positioned into the contralateral internal iliac artery via the femoral artery approach. The catheter’s side hole, located between the distal and proximal balloons, facilitated angiographic visualization of the contrast medium (CM) flow into the urinary bladder. Hemodynamic analysis of the CM in the pelvic arteries during D-BOAI was conducted using 2D-PA. Regions of interest (ROIs) were delineated at the side hole (A) as the outflow point for CM and in the vesical artery region (B). The ratio of the area under the curve (AUC) of CM at each ROI (C = B/A) was computed. The decrease in IIABP (D) following balloon occlusion was recorded at the catheter side hole. The relationship between C and D was analyzed using Pearson’s product-moment correlation coefficient.

**Results:**

A total of 32 sides from 16 patients were analyzed. The mean C value was 0.39, and the mean D value was 55.2 mmHg, while the mean IIABP post-occlusion measured 66.2 mmHg. A significant positive correlation between C and D was identified, with a correlation coefficient of 0.704 (*p* < 0.001).

**Conclusion:**

The findings demonstrate a significant positive correlation between blood perfusion volume in the vesical artery region and the reduction in IIABP following balloon occlusion.

**Relevance statement:**

Our results suggest that decreased IIABP after balloon occlusion could result in high concentrations of anticancer drugs in the vesical artery region, and favorable local tumor control in bladder cancer.

**Key Points:**

- D-BOAI chemotherapy can treat invasive bladder cancer without radical cystectomy.
- IIABP and flow persist to some extent even following double balloon occlusion.
- 2D-PA allowed quantitative evaluation of vesical arterial perfusion volume in D-BOAI.

**Graphical Abstract:**

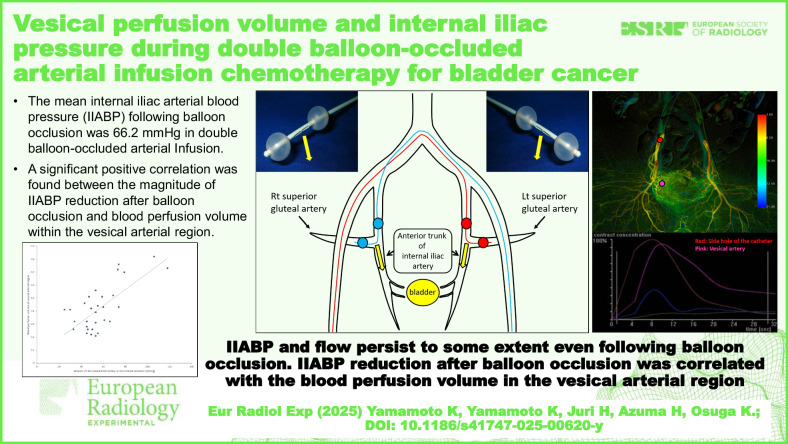

## Background

Radical cystectomy remains the standard of care treatment for muscle-invasive bladder cancer, necessitating the creation of an ileal conduit or other forms of urinary tract reconstruction. However, patient quality of life following this procedure is often significantly compromised. In recent years, advancements such as bladder reconstruction using intestinal segments such as the ileum and colon, as well as nerve-sparing cystectomy techniques to mitigate postoperative sexual dysfunction, have contributed to gradual improvements in patient outcomes. Nevertheless, these surgical interventions have long operating times and limited applicability, especially in the elderly patient population, which constitutes a substantial proportion of individuals diagnosed with bladder cancer, necessitating a different therapeutic approach.

The Osaka Medical College (OMC) regimen, developed by Azuma et al, represents an innovative approach to bladder preservation therapy and has demonstrated promising clinical efficacy [1–4]. This multimodal treatment protocol integrates transurethral resection of bladder tumors, systemic chemotherapy, radiation therapy, hemodialysis, and double-balloon-occluded arterial infusion (D-BOAI) using a four-lumen double-balloon catheter. During D-BOAI, the proximal balloon is strategically positioned at the anterior trunk of the internal iliac artery (AT-IIA), upstream of the superior gluteal artery (SGA) bifurcation, while the distal balloon is located at the origin of the SGA. This configuration isolates the AT-IIA, ensuring the targeted vesical arteries (VAs) are positioned between the balloons. The chemotherapeutic agents are then bilaterally infused through side holes located between the balloons. The OMC regimen can treat muscle-invasive bladder cancer at stage T2 or higher without radical cystectomy [1].

The utility of balloon occlusion in D-BOAI is highlighted by several mechanisms. First, occlusion of the SGA via the distal balloon effectively restricts the extracorporeal spread of anticancer drugs. The proximal balloon occludes the AT-IIA, reducing arterial blood flow to the internal iliac arterial region, thereby enhancing drug concentration within the vesical arterial territory and potentially leading to favorable local control of bladder cancer. Despite these advantages, the degree to which occlusion of the AT-IIA decreases internal iliac arterial blood pressure (IIABP) remains unclear. Furthermore, it is yet to be determined whether there is a quantifiable relationship between the reduction in IIABP caused by proximal balloon occlusion and the concentration of anticancer drugs in the vesical artery region (VA-ROI).

The advent of two-dimensional perfusion angiography (2D-PA) has facilitated the clinical quantification of blood flow using digital subtraction angiography (DSA) images. 2D-PA is a sophisticated technique that enables the quantitative analysis of tissue perfusion through postprocessing of standard DSA images, producing time–density curves that represent contrast medium dynamics within a region of interest (ROI). This technology allows for real-time assessment of perfusion in vessels distal to occlusions during D-BOAI. Although primarily employed to evaluate blood flow in endovascular recanalization procedures for peripheral arterial disease [5–10], 2D-PA has also been utilized to assess contrast medium distribution in transarterial chemoembolization (TACE) for hepatocellular carcinoma [11–16].

Although the clinical outcomes of D-BOAI have already been demonstrated by Azuma et al [1–4], the hemodynamics of the VA region during D-BOAI have not been elucidated and need to be clarified to establish the basis for the favorable clinical outcomes of D-BOAI. This study aimed to quantify the reduction in IIABP following balloon occlusion and to investigate whether there is a correlation between this reduction and the blood perfusion volume in the VA region, as measured by 2D-PA.

## Materials and methods

### Study design and patient recruitment

The study was conducted in strict adherence to the ethical principles outlined in the Declaration of Helsinki and received approval from the institutional review board of Osaka Medical and Pharmaceutical University (date: 2023.03.23, no. 2022-199). Due to its retrospective design, the requirement for written informed consent was waived. From April 2014 to October 2014, we conducted a study using D-BOAI in patients with invasive bladder cancer, who received a thorough explanation of bladder cancer treatment, including orthotopic bladder substitution. The present study also included patients with non-invasive bladder cancer who chose D-BOAI after receiving a thorough explanation about bladder cancer treatment. Eligibility criteria included a histological diagnosis of stage Ta, T1, T2, or T3 bladder cancer without evidence of distant metastasis. Sixteen patients (13 men, 3 women; median age: 63.5 years, range: 45–77 years) were successfully included and underwent postprocessing using conventional DSA and 2D-PA imaging techniques. Detailed clinical characteristics are provided in Table 1. Patients under twenty years of age were excluded.

**Table 1:** Patient characteristics

Variables	Data
Age in years, median (range)	63.5 (45–77)
Sex	
Male	13 (81)
Female	3 (19)
Clinical stage	
T-stage before	
Ta	1 (6)
T1	2 (12)
T2	10 (63)
T3	3 (19)
T4	0
Tumor histology	
UC	
G1	0
G2	3 (19)
G3	12 (75)
Unknown	1 (6)

Data are presented as *n* (%) unless otherwise indicated

*UC* Urothelial carcinoma

### BOAI procedure and imaging protocol

**Fig. 1:**
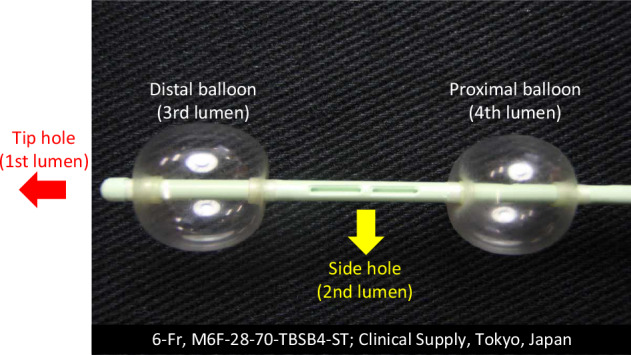
Four-lumen double-balloon catheter. The first lumen serves as the tip hole for guidewire insertion. The second lumen features a side hole for injecting an anticancer drug or contrast medium. The third and fourth lumens are used to inflate the distal and proximal balloons, respectively. The distance between the two balloons is 4 cm, and each balloon has a diameter of 12 mm. Anticancer drugs or contrast medium are administered through the side hole

The BOAI procedure utilized a 6-Fr intra-arterial catheter with dual occlusion balloons (M6F-28-70-TBSB4-ST; Clinical Supply) (Fig. 1), equipped with four distinct lumens: the first for guidewire insertion, the second for administering anticancer drugs or contrast medium, the third for inflating the distal balloon, and the fourth for inflating the proximal balloon.

**Fig. 2:**
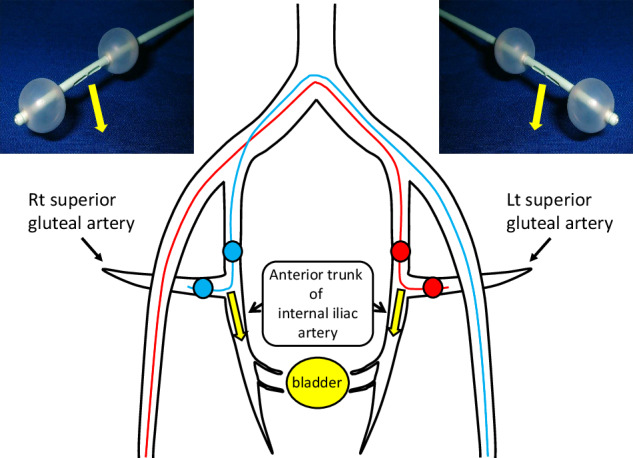
Schematic representation of D-BOAI. Double-balloon catheters (6 Fr) are inserted into the left (Lt) and right (Rt) superior gluteal arteries through the contralateral femoral arteries. The side holes, located between the distal and proximal balloons, are positioned at the origin of each VA to enable clear angiographic visualization of the flow of the injected agent into the urinary bladder

This four-lumen double-balloon catheter was inserted into the internal iliac artery via the contralateral femoral artery. Once the distal balloon traversed the bifurcation of the AT-IIA, the balloons were positioned and inflated, isolating the AT-IIA to ensure targeted delivery to the vascular territories (VAs) of interest (Fig. 2). Using conventional DSA, the correct positioning was verified, confirming the absence of contrast medium in the SGA and no backflow upstream of the AT-IIA, with clear tumor staining indicative of active flow into the urinary bladder. During intra-arterial infusion chemotherapy as part of the OMC regimen, 100 mg of cisplatin was administered locally over 1 h via the catheter’s side holes between the balloons. Prior to chemotherapy, conventional DSA imaging was conducted with both balloons inflated.

All angiographic images were acquired using the same angiography system (Artis zee BA; Siemens AG), maintaining consistent injection parameters. The angiographic protocol involved the administration of 10 mL of iopamidol (370 mg I/mL) at 1.5 mL/s, with images captured in the anteroposterior projection at 4 frames/s. The high-pressure-resistant extension tube connected to the bilateral catheters was managed through a three-way stopcock using a Mark V ProVis Angiographic Injection System (Medrad, Inc.). Because our hospital’s angiography system cannot rotate in the pelvic position due to the limited range of motion of the C-arm, cone beam CT assessment was not performed.

### Measurement of arterial blood pressure

**Fig. 3:**
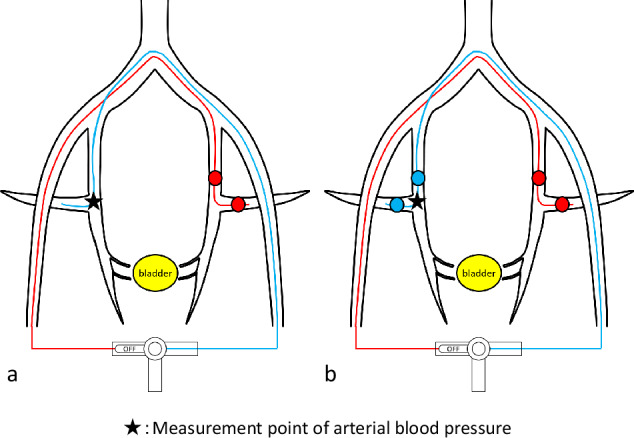
Schematic of D-BOAI illustrating preocclusion (**a**) and postocclusion (**b**) by balloons. The figure depicts arterial pressure measurements at the side hole of a catheter inserted into the right internal iliac artery, with adjustments made via a three-way stopcock. Arterial pressure is measured both before and after balloon occlusion. Similarly, pressure is measured at the side hole of the catheter placed in the left internal iliac artery, with adjustments using the three-way stopcock

Arterial blood pressure was measured using a pressure transducer (BD DTXplus; BD Bioscience). The four-lumen double-balloon catheter was bilaterally inserted via the internal iliac artery into the SGA, with the proximal balloon positioned and dilated at the AT-IIA and the distal balloon dilated at the SGA. All four balloons were dilated bilaterally. The second lumen of the four-lumen double-balloon catheter, which included the side-hole segment located between the balloons, was connected on both sides to a single extension tube through a three-way stopcock. The procedure commenced by altering the direction of the three-way stopcock to disconnect the catheter inserted into the left internal iliac artery, enabling the measurement of arterial blood pressure at the side-hole segment of the catheter inserted into the right internal iliac artery, both before and after balloon occlusion (Fig. 3a, b).

Subsequently, the stopcock direction was adjusted to disconnect the right internal iliac artery catheter, allowing for the measurement of arterial blood pressure at the side-hole segment of the catheter inserted into the left internal iliac artery, also recorded both before and after balloon occlusion.

### DSA postprocessing and measurements

**Fig. 4:**
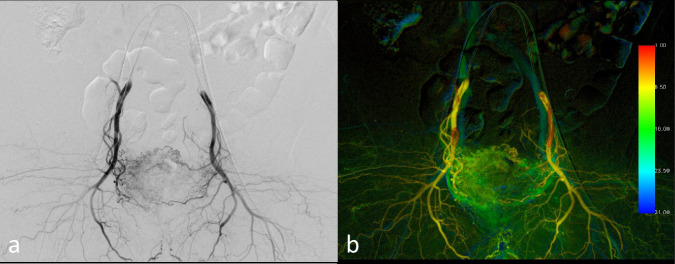
**a** DSA performed with both distal and proximal balloons inflated (D-BOAI). **b** A color-coded image created through postprocessing of the DSA image using Syngo iFlow, depicting the same patient as in **a**

DSA data acquired during D-BOAI were transferred to a dedicated image reconstruction workstation (syngo X Workplace; Siemens Healthcare) (Fig. 4). Hemodynamic parameters were analyzed using syngo X software, which provides a color-coded single image by calculating time–intensity curves for each pixel within the DSA series. This method visualizes the propagation of the contrast medium through the vascular structures. Time to peak enhancement and the area under the curve (AUC) were extracted from these pixel-specific time–intensity curves. Time to peak enhancement was represented using a color gradient (red to blue), denoting early, intermediate, and delayed flow phases, enabling evaluation of contrast medium inflow and outflow within specific pixels or ROIs. The AUC value in an ROI depicted the total volume of contrast medium flow within the series. This comprehensive, color-coded imaging technique facilitated the assessment of arterial contrast medium dynamics in a single visualization. Hemodynamic parameters for the study were evaluated based on the AUC values.

**Fig. 5:**
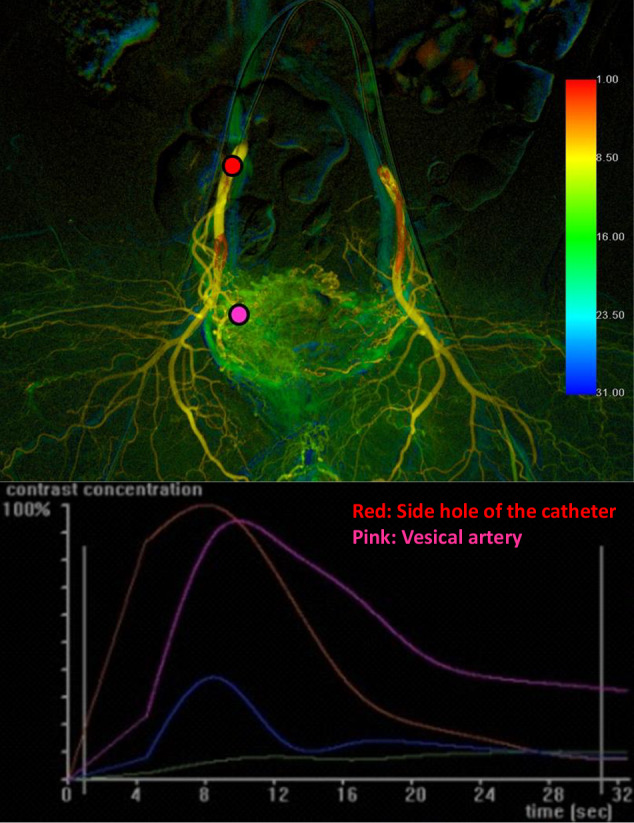
Color-coded images and corresponding time–intensity curves following contrast medium injection. Red and pink regions of interest (ROIs) were identified on the ipsilateral side of the catheter and the VA, respectively. The red ROI, located on the catheter side hole, was used as the reference point. The AUC was calculated by summing the relative density at each time point, normalized to the reference point, and dividing by the frame rate

ROIs were positioned at the catheter side hole, designated as the reference point (Ref-ROI), and within the VA-ROI on the color-coded D-BOAI image (Fig. 5). Due to the limitations of resolution in 2D-PA, including the presence of a vesical arterial anomaly or multiple VAs. The ROI was placed in the visually most densely vascularized area in the VA-ROI on the 2D-PA. Time–intensity curves were generated for each ROI and analyzed. The AUC was computed by summing the relative density of the VA-ROI compared with the Ref-ROI at each time point, normalized by the frame rate. To assess total relative perfusion, the AUC was calculated from the initial first-pass perfusion up to 25 s. This AUC was not an absolute value but a comparative metric, determined as the AUC of the VA-ROI divided by the AUC of the Ref-ROI. The comparative value (CV) for each ROI was calculated as follows:

$${\rm{CV}}={\rm{AUC}}\; {\rm{of}}\; {\rm{the}}\; {\rm{VA}}-{\rm{ROI}}/{\rm{AUC}}\; {\rm{of}}\; {\rm{the}}\; {\rm{ipsilateral}}\; {\rm{Ref}}-{\rm{ROI}}$$

### Statistical analysis

The relationship between the change in IIABP following balloon occlusion and the CV was evaluated using correlation analysis. Pearson’s product-moment correlation was employed, with a *p*-value threshold of < 0.05 considered statistically significant. All statistical analyses were performed using EZR software (Saitama Medical Center, Jichi Medical University), a graphical user interface for R (The R Foundation for Statistical Computing), specifically modified from the R Commander to enhance its utility for biostatistical applications. The Kolmogorov–Smirnov test confirmed that the values of IIABP following balloon occlusion and the CV followed normal distributions. It was confirmed that the sample size was sufficient using R.

## Results

Patient characteristics are summarized in Table 1. Table 2 presents the pre- and post-balloon occlusion IIABP values, along with the degree of IIABP reduction following balloon occlusion. The mean IIABP prior to balloon occlusion was 121.7 ± 21.4 mmHg (range: 83–168 mmHg), whereas the mean IIABP post-occlusion was 66.2 ± 15.8 mmHg (range: 30–99 mmHg). The mean decrease in IIABP following balloon occlusion was 55.2 ± 19.2 mmHg (range: 0–118 mmHg). The mean rate of IIABP reduction from pre-occlusion to post-occlusion of the balloons was 45% (range: 0–70%).

**Table 2:** IIABP

	Mean ± SD (right)	Mean ± SD (left)	Mean ± SD (total)
Pre-occlusion	121.9 ± 21.0 (range: 83–164)	121.4 ± 21.9 (range: 85–168)	121.7 ± 21.4 (range: 83–168)
Post-occlusion	65.8 ± 13.7 (range: 30–91)	66.6 ± 17.7 (range: 32–99)	66.2 ± 15.8 (range: 30–99)
Amount of decrease	56.2 ± 18.3 (range: 25–106)	54.2 ± 23.6 (range: 0–118)	55.2 ± 19.2 (range: 0–118)

Data units are presented as mmHg unless otherwise indicated

*SD* Standard deviation

**Fig. 6:**
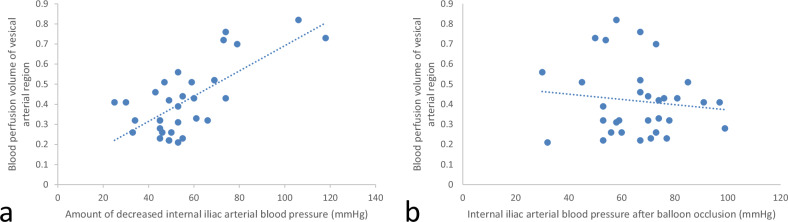
**a** Graph illustrating the correlation between the reduction in IIABP and the perfusion volume of the VA-ROI. A statistically significant positive correlation was found (*r* = 0.704, *p* < 0.001). **b** Graph showing the relationship between IIABP after balloon occlusion and perfusion volume of the VA-ROI. No statistically significant correlation was observed (*r* = -0.121, *p* = 0.511)

The association between the reduction in IIABP post-balloon occlusion and the CV is shown in Fig. 6. A statistically significant positive correlation was identified between the degree of IIABP reduction following balloon occlusion and the blood perfusion volume in the VA-ROI (*r* = 0.704, *p* < 0.001). However, no statistically significant correlation was found between post-occlusion IIABP and blood perfusion volume in the VA-ROI (*r* = -0.121, *p* = 0.511).

In the present study, none of the sixteen patients suffered Grade III or more severe toxicities with the National Cancer Institute’s Common Terminology Criteria for adverse events v5.0.

## Discussion

The present study revealed two key findings. First, the mean reduction in IIABP following balloon occlusion was quantified at 55.2 mmHg, while the mean IIABP post-occlusion measured 66.2 mmHg. Second, a significant positive correlation was identified between the magnitude of IIABP reduction after balloon occlusion and blood perfusion volume within the VA-ROI, as assessed using 2D-PA.

The primary implication of the first finding is that internal iliac artery blood pressure and flow persist to some degree even following double balloon occlusion at both the AT-IIA and the SGA. Similar to D-BOAI, selective balloon-occluded transarterial chemoembolization (B-TACE) is employed in hepatocellular carcinoma treatment to deliver high concentrations of chemotherapeutic agents directly to tumors under conditions of balloon occlusion [17]. During occlusion, the pressure gradient between the hepatic artery and the portal vein diminishes, preventing the lipiodol emulsion from flowing into the portal vein. Supporting this, Irie et al [17] demonstrated that B-TACE for hepatocellular carcinoma maintains arterial blood pressure and flow distal to the occlusion site. Furthermore, they established that effective, dense accumulation of lipiodol emulsion in hepatocellular carcinoma nodules could be achieved when the balloon-occluded arterial stump pressure was 64 mmHg or lower [17]. Although the liver is hemodynamically different from the pelvic region due to dual blood flow control of the hepatic artery and portal vein, similar to balloon-occluded arterial stump pressure after balloon occlusion in B-TACE, IIABP was observed to be maintained after balloon occlusion.

Atherosclerotic disease frequently results in the occlusion of the aorta and iliac arteries, necessitating the formation of collateral networks through systemic-systemic and systemic-visceral pathways in aortoiliac occlusive disease [18]. These collateral routes can be visualized and accurately assessed through advanced imaging modalities, such as computed tomography angiography. Although the mechanisms of collateral blood flow formation after balloon occlusion are not similar to those that develop in peripheral arterial disease and other long-term chronic occlusive diseases, similar collateral pathway development processes may occur during temporary balloon occlusion of the internal iliac artery and affect the blood perfusion at the VA-ROI in D-BOAI. The drug is expected to be delivered not only to the VA-ROI but also to non-target vessels such as the inferior gluteal artery, but these collateral vessels could prevent anticancer drugs from reaching non-target vessels. Our study demonstrated that IIABP remains sustained post-balloon occlusion in D-BOAI and that there is continued peripheral blood flow originating from the occlusion site.

Importantly, our findings established a significant positive correlation between the degree of reduction in IIABP following balloon occlusion and the volume of blood perfusion in the VA-ROI as assessed by 2D-PA. Building on previous work by Irie et al [17], which indicated that greater lipiodol emulsion accumulation occurred when balloon-occluded arterial stump pressure was 64 mmHg or lower, we hypothesized that, in the context of D-BOAI, there exists a correlation between vesical arterial blood perfusion volume (as visualized on 2D-PA) and the degree of the decrease in IIABP post-balloon occlusion. The proposed mechanism underlying the enhanced concentration of anticancer agents in the VA-ROI during D-BOAI involves reduced pelvic arterial flow in the occluded state compared with non-occluded conditions. Specifically, when anticancer drugs are administered while the balloon is inflated, the reduction in pelvic blood flow allows the drugs to remain localized at higher concentrations within the vesical region, thereby potentiating their antitumor efficacy. Irie et al [17] demonstrated a similar correlation in B-TACE for hepatocellular carcinoma, wherein decreased arterial pressure due to balloon occlusion was associated with increased lipiodol emulsion deposition within the tumor. In line with these findings, our study confirmed a significant positive correlation between the reduction in IIABP post-balloon occlusion and vesical arterial perfusion volume on 2D-PA.

In this study, 2D-PA was employed to assess perfusion volume within the VA-ROI. Iodinated contrast angiography, a standard method utilized in angiographic suites, serves as a practical and intuitive compound for perfusion assessment across a variety of interventional procedures. However, conventional angiography relies on the visual assessment of arterial flow, which is inherently subjective and prone to both inter-observer and intra-observer variability. In contrast, 2D-PA represents an innovative technique for the quantitative assessment of blood flow and tissue perfusion, utilizing advanced postprocessing of standard DSA images. This method has previously demonstrated efficacy in quantifying perfusion changes in balloon pulmonary angioplasty [19], TACE, and endovascular recanalization for peripheral arterial disease, including renal and mesenteric arteries [5–10, 20–22]. By generating signal intensity curves based on the flow of contrast medium through a defined ROI, 2D-PA provides objective and quantitative information, facilitating the assessment of perfusion changes. Yamamoto et al quantitatively compared vesical arterial perfusion between single balloon-occluded arterial infusion (S-BOAI) and D-BOAI, reporting that the contrast medium in the VAs was over twice as high with D-BOAI compared with S-BOAI (right side: 3.03-fold; left side: 2.81-fold) [23].

This study has several limitations. First, the retrospective design and small sample size from a single institution may limit the generalizability of the findings. Second, although a significant correlation between the decrease in IIABP following balloon occlusion and perfusion volume in the VA region using 2D-PA was observed, a causal relationship with therapeutic efficacy and clinical outcomes was not established. Third, while the study demonstrated that IIABP and arterial blood flow are maintained despite balloon occlusion, the underlying mechanisms remain unclear. Fourth, 2D-PA is restricted to assessing blood flow within the imaging timeframe of DSA and lacks the capability to evaluate three-dimensional blood flow. Fifth, in the present study, a significant correlation was observed between the decrease in IIABP after balloon occlusion and the amount of perfusion in the bladder artery region using 2D-PA, but the actual drug concentration of the infused anticancer drug was not measured. Sixth, there is a difference between the injection rate of contrast medium in DSA and the injection rate of anticancer drug in D-BOAI, and DSA images do not accurately reflect the actual distribution of anticancer drugs to the vesical region. Seventh, the development of collateral pathways in patients with preexisting arterial disease and its effect on VA in D-BOAI has not been analyzed. Eighth, in the present study, it is not clear how this correlation directly affects patient indications, treatment efficacy, and procedure planning, and these aspects need to be clarified with additional case numbers in the future.

In conclusion, the present study revealed that the mean decrease in IIABP following balloon occlusion was 55.2 mmHg, and the mean IIABP post-occlusion was 66.2 mmHg. Additionally, a significant positive correlation was identified between the degree of IIABP reduction and perfusion volume in the VA-ROI as measured by 2D-PA. Local tumor control in D-BOAI is presumably due to high accumulation of anticancer drugs in the VA-ROI, the only imaging tool to confirm whether anticancer drugs accumulate in the VA-ROI before D-BOAI procedure is DSA and 2D-PA. The results of the present study suggest a partial mechanism leading to the high accumulation of anticancer drugs in the VA-ROI before D-BOAI.

## Data Availability

The datasets used and/or analyzed during the current study are available from the corresponding author on reasonable request.

## Abbreviations

2D-PA: Two-dimensional perfusion angiography
AT-IIA: Anterior trunk of the internal iliac artery
AUC: Area under the curve
B-TACE: Balloon-occluded transarterial chemoembolization
D-BOAI: Double balloon-occluded arterial infusion
DSA: Digital subtraction angiography
IIABP: Internal iliac arterial blood pressure
ROI: Region of interest
SGA: Superior gluteal artery
VA: Vesical artery
